# Functional echolalia in autism speech: Verbal formulae and repeated prior utterances as communicative and cognitive strategies

**DOI:** 10.3389/fpsyg.2023.1010615

**Published:** 2023-02-23

**Authors:** Fan Xie, Esther Pascual, Todd Oakley

**Affiliations:** ^1^School of Foreign Languages, Guangdong University of Technology, Guangzhou, China; ^2^Institute of Linguistics, Shanghai International Studies University, Shanghai, China; ^3^Department of Cognitive Science, Case Western Reserve University, Cleveland, OH, United States

**Keywords:** autism spectrum disorder, linguistic units, socio-communicative formulae, socio-cultural emblems, function, elicitation

## Abstract

Echolalia, the echoing of prior speech, is a typical characteristic of autism. Long considered meaningless repetition to be avoided, echolalia may in fact be used functionally in autism. This paper explores the functions of echolalia by children with autism. Based on two prior studies, we designed an elicitation task involving images of 12 professions (teacher) and 12 objects (birthday cake) commonly associated with given conventionalized expressions in Mandarin (e.g., “*sheng ri kuai le!*” ‘Happy birthday!’). Eight Chinese children with autism (mean age: 55.50 ± 8.64) were asked to name and describe these images. All our participants produced a relatively high proportion of echolalia, mostly for naming, description, and topic development, a small percentage being used as conversation maintenance strategy or as cognitive strategy. This indicates that echolalia is often used communicatively in autism speech.

## Introduction

Autism Spectrum Disorder (henceforth ‘ASD’) is an increasingly common neurodevelopmental condition currently affecting between 1 and 2% of the population in North America, Europe, and Asia (Chien et al., 2011; Parner et al., 2011; Zablotsky et al., 2015), including mainland China (Sun et al., 2015). According to the latest edition of the *Diagnostic and Statistical Manual of Mental Disorders* (*DSM-5*; American Psychiatric Association, 2013), ASD is characterized by impairments or abnormalities in social interaction, repetitive or restrictive behavioral patterns and interests, as well as deficiencies in communication.

A typical characteristic of ASD, shown in 75–80% of verbal individuals, is so-called ‘echolalia’, the echo-like repetition of previously heard or spoken speech (Kanner, 1946; Prizant, 1983; Prizant and Rydell, 1984; Neely et al., 2016). Echolalia in autism has been widely documented and researched, including the investigation of its main types and functions (Fay, 1967; Prizant and Rydell, 1984; Schuler and Prizant, 1985; Cohn et al., 2022). The repetition in question may occur right after what is echoed or after some delay (i.e., immediate vs. delayed echolalia; Prizant and Duchan, 1981; Wootton, 1999), and the original utterance may be repeated entirely or partially (i.e., exact vs. mitigated echolalia; Fay and Butler, 1968). Mitigated echolalia includes occurrences in which the echoed bit appears modified (if ever so slightly) and those that are verbatim but integrated into a larger grammatical structure [see a systematic review in Neely et al. (2016)].

Echolalia has traditionally been defined as the socially awkward or inappropriate repetition of a prior utterance (or part of one) with no communicative function (Karmali et al., 2005; Valentino et al., 2012). Numerous early autism studies equated echolalia as stereotypy, a sign of cognitive impairment, and a pathological default to be discouraged, consisting of meaningless, obsessive repetition. By contrast, recent research shows that echolalia may in fact be an effective adaptive communicative strategy (Prizant, 1983; Dobinson et al., 2003; Stribling et al., 2007; Roberts, 2014; Dornelas and Pascual, 2016; Pascual and Sandler, 2016:323–342; Pascual et al., 2017), some calling for a redefinition of echolalia as an interactional resource in autism communication (Sterponi and Shankey, 2014). Examples are saying “Goal!” for ‘soccer’, quoting somebody’s words to refer to them.

In the language acquisition literature, imitation and repetition are viewed as acquisition strategies (Nelson, 1973). Using verbatim phrases may provide some young children with an alternative acquisition route into multiword speech (Lieven et al., 1992; Plunkett, 1993; Wray and Perkins, 2000). Research suggests that children with ASD use echoed repetitions much more frequently and widely, and for a longer period, than younger typically developing children (e.g., Dobinson et al., 2003; Dornelas, 2018). Moreover, while children with ASD use echolalia as an adaptive communicative strategy, controls of the same chronological age use it as rhetorical strategy, such as for humor or engagement (Pascual et al., 2017). The pathological aspect of echolalia is the degree to which it persists as dominant strategy in ASD speech compared to that of neurotypical toddlers. However, echolalia still seems to be a transitional phase that they move through as they develop other functional language skills (Kanner, 1943; Prizant, 1987). The frequency of echolalic speech in autism may predict interactional functions in communication (Dobinson et al., 2003) and even higher verbal functioning with age (e.g., Lovaas et al., 1973). Furthermore, the use of echolalia seems to reflect developmental progress in spontaneous speech and comprehension (e.g., Fay, 1967; Fay and Coleman, 1977). The trend is clearly towards considering echolalia a developmental phenomenon in the child’s normal cognitive and linguistic maturation (see overviews in Schuler and Prizant, 1985; Sterponi and Shankey, 2014).

Regarding its types, functional, interactive *immediate* echolalia may involve: turn-taking, declarative utterances, yes/no answers, and requests (Prizant and Duchan, 1981; Sterponi et al., 2014; Sterponi and de Kirby, 2016). Functional interactive *delayed* echolalia may include: turn-taking, information-providing, labeling, calling, protesting, requesting, completing, affirming, directing, and maintaining social interaction [Prizant, 1983; Prizant and Rydell, 1984; Pascual et al., 2017, for an overview see Schuler and Prizant (1985) and Volkmar et al. (2005)]. There is however still little understanding of the underlying cognitive and functional underpinnings of echolalia. Most prior studies focus on immediate echolalia, which is easier to identify and interpret (echoing part or all of the question immediately within the conversation turn, e.g., Examiner: “Is the Mommy holding the baby?”; Child: “Mommy is holding the baby,” Paccia and Curcio, 1982, p: 44). This leads to a partial and piecemeal understanding of this functionally complex phenomenon. Given the specificity of repetitive speech in ASD, research on delayed echolalia consists predominantly of qualitative analysis of spontaneous speech in naturalistic settings (e.g., Dobinson et al., 2003; Stribling et al., 2007; Santen et al., 2013).

The research reported here is based on a prior naturalistic study of echolalia in autism conversation, with two control groups matched with the autism group in mental and chronological age, respectively, (Pascual et al., 2017) and a follow-up elicitation study, also with two control groups (Dornelas, 2018). In these two studies, all the usages of echolalia by the autism group were functional, and they were all understood by their interlocutors. The difference in the use of echolalia between the autism and the young typically developing group was principally one of quantity (the autism group producing less creative speech), both groups using it as a communicative strategy for similar functions.

In this paper, we study the functional complexity of echolalia in autism speech using an elicitation task to deepen our understanding of how this pervasive phenomenon functions in standardized contexts. Hence, our goal is not to compare neurotypical to neurodivergent speakers, but to explore how children with ASD use echolalia as a communicative strategy to answer simple questions or manage a conversation. We focus on the production of echoed verbal formulae that are not specific of a given child but widespread in the linguistic community at large. We address the following research question: Do Chinese children with ASD produce echolalic utterances as functional strategies or as pathological stereotypy or both? If echolalia is used functionally by ASD children, then how do they manage a conversation, given their limited resources, by using this strategy? By combining qualitative and quantitative methods in the analysis of elicited language data, we aim to examine the forms and functions of echolalia, its most common sources, and its proportion vis-à-vis referential and descriptive alternatives.

As for the criteria for identification of echoed utterances, we adopted the fundamental definition of echolalia as involving previously encountered word strings. We followed the standard distinguishing characteristics used in prior studies (Peters, 1983; Wray and Namba, 2003; Pascual et al., 2017), relying on multi-modal cues (e.g., rising intonation, eye contact, gestures, posture, voice or sound imitation), as well as corroborating evidence both from the context (co-text and metapragmatic information), and the parents’ and/or the therapist’s informed interpretations. The determination of degree of functionality was equally based on multi-modal and contextual cues, also asking the parent or therapist for their interpretation when needed. These criteria supported the coders’ native speaker judgments.

## Methodology

### Participants

Eight Mandarin-speaking preschool children with ASD, aged from 3 to 6 years old (mean age = 55.50 ± 8.64), were recruited from the Zhejiang provincial children’s early intervention center *Green Apple Home* in Hangzhou, China. These involved two cases of what could be considered ‘severe’ autism (children 3 and 5), four ‘moderate’ cases (children 2, 4, 6, and 8), and two ‘mild’ cases (children 1 and 7). All these children had been previously diagnosed by experienced child psychiatrists and had met the diagnostic criteria of the latest edition of the *Diagnostic and Statistical Manual of Mental Disorders* (*DSM-5*, American Psychiatric Association, 2013). We supplemented their autism diagnosis with the Chinese version of the *Autism Behavior Checklist* (ABC; Yang et al., 1993). The ABC is one of the most frequently used scientific screening assessments in studies of ASD in mainland China (Sun et al., 2013; Su et al., 2014, 2018). The higher a child’s ABC score, the higher their level of impairment. According to the Chinese version of the ABC test, individuals with a total score of 62 or higher are highly likely to suffer from ASD, the cut-off score being 31. This helps distinguish children who are questionably autistic from those unlikely to be autistic (Yang et al., 1993). The data show that our participants’ average ABC score is 49.13 (SD = 12.98), ranging from 31 to 65, which confirms that all our 8 participants unequivocally met the diagnostic criteria of autism.

We excluded children with ASD who did not finish the elicitation task and those whose parents did not complete the parental report (i.e., the ‘Putonghua Communicative Development Inventory’; Tardif et al., 2008). Eight children were selected from a pool of 63 children with ASD, because they were echolalic and produced relatively abundant language data on average. The reason why only eight participants were tested is that the pragmatic and child-specific nature of the phenomenon under study requires close observation of the data for metalinguistic cues as well as numerous and lengthy consultations with parents and therapists. Additionally, only boys were included because there was a preponderance of boys compared with girls in our larger pool (50 boys vs. 13 girls), which is representative of the average sex distribution in the autism population.

The basic information of the participants is shown in Table 1, which contains their age in months, length of therapy time, total vocabulary scores on the PCDI test, and the age of vocabulary-matched typically developing children. Following the norms established in Tardif et al. (2008), the PCDI vocabulary production scores of these eight Chinese boys with ASD can be matched to typically developing Chinese boys at 25 months of age, as shown in Table 1 (vocabulary production scores: ASD: 607 ± 175.47 vs. Typically Developing 25 months: 609 ± 224, *t* = 0.194, *p* = 0.849 > 0.05, *d* = 13).

**Table 1:** Characteristics of our male participants with ASD and the matched typically developing (TD) boys in Tardif et al. (2008).

ASD (*n* = 8)	Age in months	Therapy time length (in months)	PCDI vocabulary production scores in ASD	PCDI vocabulary production scores in matched TD children in the norms by Tardif et al. (2008) (*n* = 35)
Mean	55.50	17.13	607	TD 25 months: 609
(SD)	(8.64)	(9.70)	(175.47)	(224)
Range	47–70	3–24	227–779	241–781

We also assessed general vocabulary size and grammatical competence by having the children’s parents complete the ‘Putonghua Communicative Development Inventory’, specifically the sub-scale ‘Putonghua Communicative Development Inventory: Words and Sentences’ (Tardif et al., 2008), which is the Chinese version of the ‘MacArthur-Bates Communicative Development Inventory’ (CDI, Fenson et al., 1993). Table 2 shows that open class words (i.e., nouns and verbs), which are useful in an elicitation task like the one in this study, are more accessible to our participants than closed class ones (i.e., pronouns, classifiers, and question words).

**Table 2:** PCDI vocabulary production scores.

Five lexical subcategories					
ASD (*n* = 8)	Nouns (*n* = 371 items)	Verbs (*n* = 194 items)	Pronouns (*n* = 24 items)	Classifiers (*n* = 20 items)	Question words (*n* = 12 items)
Mean (SD)	317.88 (63.55)	133.63 (55.48)	11.75 (8.26)	11.63 (6.61)	7 (4.57)
Range	180–368	20–190	0–23	0–20	0–12

Table 3 presents the children’s mean utterance length and sentence complexity in comparison with the norm. This reveals no significant difference between our participants, averaging 55 months, and vocabulary-matched controls at 25 months of age (*p* = 0.630 > 0.05).

**Table 3:** Mean length of utterance (SD) and sentence complexity scores of our male participants with ASD and the matched typically developing (TD) boys in Tardif et al. (2008).

Grammatical categories	ASD 55.5 months (*n* = 8)	TD 25 months (*n* = 35)	*t*	d
Mean length of utterance	6.29 (3.61)	–	–	–
Sentence complexity (*n* = 27)	55.13 (24.71)	51.9 (22.9)	1.353	26

Thus, while our eight participants possess a robust vocabulary and well-developed and complex sentence structure when compared to low-verbal children with ASD, they still lag behind their vocabulary-matched typically developing toddlers for an average of 30 months.

### Visual stimuli

To ensure that our elicitation material was recognizable to all participants vis-à-vis their autism diagnosis, age, and socio-cultural background, we first selected 50 potential visual stimuli. These were chosen based on the ‘Putonghua Communicative Development Inventory’ vocabulary checklist (Tardif et al., 2008), together with the results of two studies on functional echolalia by Brazilian children with ASD (Pascual et al., 2017; Dornelas, 2018). These 50 images were presented to 175 Chinese parents of children with ASD, who scored each according to their children’s familiarity with the referents and recognition of the images using a Likert scale.

**Figure 1:**
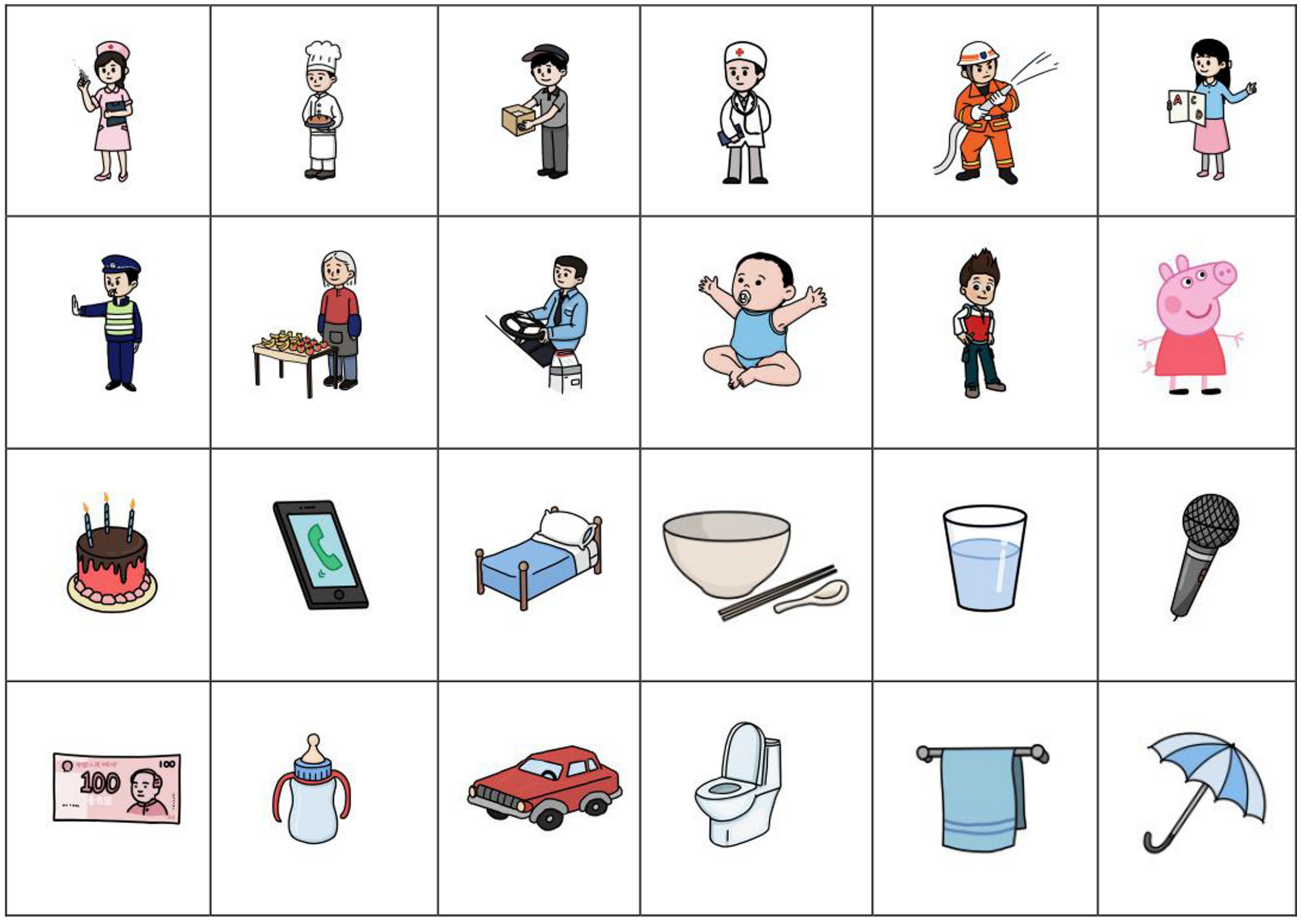
Visual stimuli.

Based on this parental pre-test, we selected 12 professions (e.g., nurse) or (types of) individuals (e.g., baby) and 12 entities (e.g., birthday cake) with which the children were most familiar and that are commonly associated with fixed expressions in Mandarin Chinese (e.g., “*da zhen!*” ‘Give an injection!’, for the nurse; “*sheng ri kuai le!*” ‘Happy birthday!’ for the cake), see Figure 1 (All figures are found in the Supplementary materials folder).

A five-score Likert scale test on the children’s familiarity with the concepts and level of recognition of these designed images was filled in by the parents after the task, to prevent data contamination. Our eight participants were reported to be quite familiar with the concepts (Mean = 4.10, SD = 0.47) and to be able to recognize the images correctly (Mean = 4.14, SD = 0.44).

### Protocol

The visual stimuli were presented by the first author on an iPad. All stimuli were randomized automatically in four different sequences, with no images from the same category appearing continuously. On each image, participants were asked: “*zhe shi shen me?*” (‘What’s this?’) or “*ta shi shei?*” (‘Who’s this?’) and “*zhe ge ke yi yong lai gan shen me?*” (‘What’s it used for?’) or “*ta hui gan shen me?*” (‘What does s/he do?’). An average of 5 s was allowed between the first appearance of an image and the first question to allow time for the children to shift attention and focus on the new picture.

The entire task was video and audio recorded in a quiet room in the center where the children receive daily therapy and took an average of over 14 min, the total time for all participants being 115 min and 35 s. A parent was present during the entire task, sitting approximately three meters from the child, to put the child at ease and facilitate querying about ambiguous or hard-to-interpret utterances of their children. These consultations were also recorded.

## Qualitative analysis

In our data, echoes of previously heard enunciations were mostly used functionally, being either question oriented or conversation oriented. Some echoed occurrences related to the images were produced before the experimenter’s question and were thus free associations coded as semi-functional echolalia. We encountered two echoed utterances used for no apparent communicative or cognitive function, and thus coded as non-functional echolalia.

We encountered numerous instances of delayed functional echolalia used to answer the experimenter’s questions (i.e., ‘Question oriented’). The main forms of such functional echolalia were: (i) conventional linguistic units [i.e., socio-communicative formulae and socio-cultural emblems, see Pascual et al. (2017)]; (ii) specific prior enunciations (i.e., lines from songs or movies, or the child’s own life); and (iii) onomatopoeia (e.g., imitations of an object’s sound).^1^ The main functions of fixed verbal formulae used as question-oriented echolalia were: (i) naming (e.g., “*sheng ri kuai le!*” ‘Happy birthday!’ to name the birthday cake); (ii) description (e.g., “*shui jiao*” ‘Go to sleep’ to describe the function of a bed); and (iii) topic development (e.g., “*bi sai kai shi! bi sai!*” ‘The competition begins! Let us compete!’ to expand on the child’s prior answers on a car’s name and function). Second, we encountered instances of immediate functional echolalia, in which the child repeated the experimenter’s question in the preceding turn. These seemed to be used as: (i) a conversation management strategy (i.e., ‘Conversation oriented’, e.g., “*ta shi shei?*” ‘Who’s this?’ – “*ta shi shei?*” ‘Who’s this?’, the experimenter’s question being repeated to show conversation engagement); or as (ii) a cognitive strategy, namely a type of inner speech to organize one’s thoughts or aid cognitive processing (i.e., ‘Self-oriented’, e.g., “*ta shi shei?*” ‘Who’s this?’ – “*ta shi shei? ta shi chu shi*” ‘Who’s this? This is a chef’, an immediate repetition produced when searching for the correct answer).

We now discuss specific echolalic occurrences in our data, divided into: (i) delayed functional echolalia used to answer questions; (ii) immediate functional echolalia used as conversation management or cognitive strategy; and (iii) semi-functional echolalia, in free associations, as well as non-functional echolalia, when no relation could be found between the echoed utterance and the target image.

### Delayed functional echolalia: Question oriented

As expected, our participants relied on delayed echolalia to answer the experimenter’s questions in the elicitation task. Consider first this response on the image for ‘toilet’ (echoed utterances in examples are underlined):

(1) Child 5, Resp. 18: Toilet.
Experimenter: 这是什么?
*zhe shi shen me?*
What’s this?
Child: 上厕所。
*shang ce suo.*
Go to the restroom/Go pee or poop.

In (1), the child does not provide the name of the image asked about. Instead, he produces the commonly recognized Chinese verbal expression that young children and caregivers usually use to indicate the need to relieve oneself. Interestingly, even though the child knew the correct name, he instead produced the echoed formula including this noun. This seems to show that the name might not be stored in memory in isolation, but be more easily retrievable as part of a whole linguistic unit, i.e., a verbal formula associated with a speech act related to the referent. A similar example is:

(2) Child 3, Resp. 1: Mobile phone.
Experimenter: 这是什么?
*zhe shi shen me?*
What’s this?
Child: 给妈妈打电话。
*gei mama da dian hua.*
Make a call to Mommy.

Here, the child uses a conventionalized and widely recognized verbal expression for making a phone call to name a mobile phone. Together with this socio-communicative formula, echoed from conversations (over) heard repeatedly in his daily life, the child adds information on the specific person to be called. This verbal formula was also used to name this image by Child 5 (Resp. 22) and was freely associated with the phone by children 7 and 8. It was used for topic development by Child 1 after providing the correct name for ‘mobile phone’, also as mitigated echolalia, adding variations in the person to be called (“*da dian hua gei ba ba*” ‘Make a call to Daddy’, Resp. 7).

The second function of question-oriented echolalia in our data was description, which occurred as an answer to the second question. Consider first:

(3) Child 6, Resp. 6: Bowl.
Experimenter: 这个可以用来干什么?
*zhe ge ke yi yong lai gan shen me?*
What’s it used for?
Child: 吃意大利面。
*chi yi da li mian*.
(It’s used to) Eat spaghetti.

This response constitutes an echoed specific prior interaction used to describe the bowl’s function, evidencing the child’s prior experiences with bowls. According to the child’s mother, this utterance was reenacted from the ‘*Peppa Pig eats spaghetti*’ episode from the popular animated television series. This expression is thus not creative but echolalic. It coincides with the exact utterance from that cartoon and it cannot be a spontaneous description of the target image, for no spaghetti is pictured in it. Apparently, this child loves eating spaghetti and he frequently uses this expression to request spaghetti or Chinese noodles when seeing a bowl or plate in his daily life. A similar strategy is illustrated in:

(4) Child 1, Resp. 23: Firefighter.
Experimenter: 他会干什么?
*ta hui gan shen me?*
What does he do?
Child: 宝宝, 那是我的宝宝, 那是我的宝宝! 那是我的哥哥。
喷水, 喷水! 发射, 把火泼灭!
*bao bao, na shi wo de bao bao, na shi wo de bao bao! na shi*
*wo de ge ge. pen shui, pen shui! fa shei, ba huo po mie!*
Baby, that’s my baby, that’s my baby! [Pause-2 s].
That’s my older brother. [Pause-5 s].
Spray water, spray water! Shooting, (let us) put out the fire!

Here the child theatrically reenacts a dramatic firefighting scene, using different voices. According to his mother, the child repeated part of the dialogues from a firefighter cartoon movie. The first two interjections are ascribed to two eyewitnesses: the parent and the younger sibling of two fire victims. The child then shifts voice to the rescuer, shouting the standard fixed expression Chinese firefighters typically use when calling for rapid, joint action (“*pen shui…!*,” ‘Spray water*…!*’). These utterances serve to describe the firefighter’s job by lively demonstrating the different vocal registers of victims and first-responders.

During the task, our ASD participants also echoed speech to expand on the topic of the ongoing conversation. These cases of topic development followed the correct naming or description of the functions of the objects or professions of individuals, further describing them vividly, often with an animated tone, iconic gestures (*cf.* Huang et al., 2020), or facial expressions. Such expansions were always related to the children’s direct experience of using the target entities or interacting with the target professionals or individuals, as confirmed by the children’s parents. Consider first:

(5) Child 1, Resp. 4: Chef.
Experimenter: 他会干什么?
*ta hui gan shen me?*
What does he do?
Child: 蔬菜, 面包。他会煮一下, 菜。汉堡, 做好咯！
*shu cai, mian bao. ta hui zhu yi xia, cai. Han bao, zuo hao*
*luo!*
Vegetables, bread. He can cook, [Pause: 14 s] vegetables. [Pause: 9 s]
[Iconic gesture].The hamburger, [Pause: 2 s] is ready!’ [Iconic gesture].

In (5), the child first answers what a chef does by providing two types of food typically prepared by such professionals. He subsequently gives a descriptive statement about chefs cooking, simultaneously making iconic cooking gestures. The child then takes the chef’s voice to say something like ‘Order up!’, indicating that a hamburger is ready for consumption, with both hands up in the iconic gesture of delivering food to customers. This can be regarded as an expansion on the topic of what a chef masters, further illustrating this profession. According to the child’s mother, he echoed the exact words related to a chef that he had heard on a televised cartoon series. The child associated the chef with the socio-communicative formula that was part of the overall social situation represented in a restaurant scene in the original cartoon.

A similar example is:

(6) Child 6, Resp. 10: Policeman.
[Responding before question].
Child: 保安叔叔, 抓住别人的。别跑, 把你抓走!
*bao an shu shu, zhua zhu bie ren de. bie pao, ba ni zhua zou！*
Security uncle [agent], catches someone. [Pause: 4 s].
Do not run, (I’ll) take you away!

Here, the child directly names the profession presented, mistaking the policeman for a community security agent. After naming what he saw in the picture, the child utters a descriptive statement on what the police do, namely arrest people. Then, after a pause, he expands on it by taking the voice of a policeman addressing a suspect. This occurred before any questions were asked, suggesting that the child anticipated the experimenter’s questions and interiorized their order (‘Who’s this?’ and ‘What does s/he do?’). This is plausible, this being the tenth target image and appearing after he provided the correct answers to the anticipated test questions for images 4, 6, and 8. After responding to the anticipated questions, the child develops the topic by reenacting verbatim an enunciation from a cartoon on police agents that he loves, according to the child’s mother.

We now discuss echolalic occurrences for maintaining the ongoing interaction or managing the child’s own thoughts.

### Immediate functional echolalia: Conversation oriented and self-oriented

Except for children 1 and 4, all our participants occasionally repeated (part of) the experimenter’s question in the preceding turn. Immediate echolalia seemed invariably used either to show engagement or to manage their own thought processes. Consider first this piece of dialogue:

(7) Child 3, Resp. 13: Teacher.
Experimenter: 他是谁?
*ta shi shei?*
Who’s this?
Child: 他是谁?
*ta shi shei?*
Who’s this?’
Experimenter: 他会干什么?
*ta hui gan shen me?*
What does she do?
Child: 他是谁?
*ta shi shei?*
Who’s this?’

Here, the child repeats exactly what the experimenter had just asked, repeating this first question again after the second question. The child does not provide an answer to either question, while looking committed to the task. His steady gaze on the test image shows full focus, his calm facial expression indicating that he did not repeat the experimenter’s words out of impatience or annoyance. Instead, the child indicated his wish to keep the conversation going. This same child repeated the immediately preceding test questions with a similar focused and calm demeanor without managing to provide the right answers for other stimuli (responses 11, 14, 22, and 23). A similar example from a different child is:

(8) Child 8, Resp. 21: Baby bottle.
Experimenter: 这是什么?
*zhe shi shen me?*
What’s this?
Child: 奶瓶是用来拿调羹泡奶。
*nai ping shi yong lai na tiao geng pao nai.*
The nursing bottle is used to take a spoon to make milk.
Experimenter: 这个可以用来干什么?
*zhe ge ke yi yong lai gan shen me?*
What’s it used for?
Child: 这个可以用来干什么?
*zhe ge ke yi yong lai gan shen me?*
What’s it used for?
Experimenter: 你告诉老师, 这个可以用来干什么?
*ni gao su lao shi, zhe ge ke yi yong lai gan shen me?*
Please tell the teacher [me], What’s it used for?’
Child: 老师, 这个可以用来干什么?
*lao shi, zhe ge ke yi yong lai gan shen me?*
Miss, what’s it used for?’

In (8), when asked what baby bottles are for, the child repeats the experimenter’s question verbatim. This question is then asked once more, and the child repeats the exact question right after again, never providing the right answer. The child may have been puzzled by being asked this question, since he had already provided the proper function of the baby bottle in a recently produced turn. The child probably repeated the question to express his wish to move on to the next target image, since he looked impatient and tried to slip to the next page with his finger, keeping his gaze on the iPad without making eye contact with the experimenter. The child’s restlessness may have been exacerbated by the fact that this was the 21st visual stimulus (out of 24) that he was presented with.

The other function of immediate echolalia in our dataset was that of cognitive strategy, in which the experimenter’s prior question was repeated as a form of self-talk. Take first:

(9) Child 2, Resp. 7: Fruit Seller.
[Responding before question].
Child: 这(是)什么?不知道。
*zhe (shi) shen me? bu zhi dao.*
What(‘s) this? (I) do not know.
Experimenter: 他是谁?
*ta shi shei?*
Who’s this?
Child: 这是老妖婆。
*zhe shi lao yao po.*
This is an old witch-lady.
Experimenter: 她会干什么?
*ta hui gan shen me?*
What does she do?
Child: 是奶奶。她是买水果。
*shi nai nai. ta shi mai shui guo.*
Is grandmother. She is *[we’ll now go]* buy fruits.

Upon seeing the image of the fruit seller, the child first anticipates the experimenter’s question and echoes it, then proceeding to answer it himself. After this two-turn private dialogue with himself, and after the experimenter’s actual question, the child first answers with a delayed echoed phrase from a television cartoon series (“*lao yao po*” ‘an old witch-lady’), which according to the child’s mother was echoed from the nursery rhyme *Monk Tang rides a horse*. Subsequently, after the second question on the woman’s profession, the child produced a declarative, using the polite way to refer to an elderly woman in Chinese instead (“*shi nai nai*” ‘is grandmother’). When the experimenter asked the second question again, the child did provide the right profession through an echolalic enunciation that translates as ‘She is *[we’ll now go]* buy fruit’. The child’s initial inner question-answer pair thus seems to have helped him to think, as all and only occurrences of self-talk were followed by short pauses, and it eventually also seems to have helped him to produce acceptable answers to both questions in later turns. Alternatively, the child may have been able to provide that answer after focusing more on the test image, or the script may have been activated more slowly than others.

The following small dialogue further illustrates the use of immediate echolalia as cognitive strategy:

(10) Child 8, Resp. 10: Policeman.
Experimenter: 他是谁?
*ta shi shei?*
Who’s this?
Child: 老师, 他是谁?
*lao shi, ta shi shei?*
Miss, who’s this?’
Experimenter: 他是谁?
*ta shi shei?*
Who’s this?
Child: 他是警察。他是警察抓小偷。
*ta shi jing cha. ta shi jing cha zhua xiao tou.* […].
He’s a policeman. He’s a policeman catching a thief. […].
Experimenter: 他会干什么?
*ta hui gan shen me?*
What does he do?
Child: 他会干什么呢?
*ta hui gan shen me ne?*
What does he do?
Experimenter: 他会?
*ta hui*…?
He can [is able to] …?’
Child: 他会抓坏人。
*ta hui zhua huai ren.* […].

He can [is able to] catch bad guys. […].

Here, the child first repeats the experimenter’s immediately preceding question, using a Mandarin respectful vocative to address the experimenter. He is then asked to name the target image again, and this time the child provides the right answer, also giving information on the policeman’s profession. Regarding the second question, the child repeats exactly the experimenter’s query, the experimenter then asking it again as a leading question. This time the child quickly provides the correct answer on what a policeman does.

The repetitions of the test questions in (10) are typical instances of echolalia used as cognitive strategy. This was a common occurrence in our data, the right answer always following a repetition of the prior question when coded as cognitive strategy. In all such cases, the child was calm and speaking in a flat tone, seemingly trying to organize his own thoughts, thus using self-talk for lexical retrieval.

Both delayed and immediate echolalia were primarily used functionally by our participants, but we also found a few instances of semi-functional and non-functional echolalia.

### Semi-functional and non-functional echolalia

All occurrences of semi-functional and non-functional echolalia in our data were of delayed echolalia. Semi-functional echoed utterances were liminal cases of free association, mainly produced before the experimenter’s first question during the initial seconds after the child was presented with an image for the first time. Consider first:

(11) Child 7, Resp. 5: Delivery man.
[Responding before question].
Child: 包裹。
*bao guo.*
Parcels.
Child: 你的包裹。
*ni de bao guo.*
(Here are) your parcels.

Upon seeing the image of the delivery man, the child utters the Mandarin word for ‘parcel’. Immediately after that, the child produces a declarative formulaic expression ascribed to the delivery man, which is commonly used by delivery agents handing packages to customers. The child’s father explained that the child is familiar with the scene of picking up mail deliveries, and that in (11) he was echoing the standard Mandarin formula commonly used by delivery agents when handing packages to customers. This socio-communicative formula was not used to answer any question but was instead freely associated with the target image and thus coded as semi-functional.

Consider a similar example of semi-functional echolalia:

(12) Child 4, Resp. 9: Peppa Pig.
[Responding before question].
Child 佩奇是大的, 佩奇是小的。
*pei qi shi da de, pei qi shi xiao de*.
Peppa is big, Peppa is small.
Experimenter: 她是谁?
*ta shi shei?*
Who’s this?
Child: 她是佩奇。
*ta shi pei qi.*
This is Peppa.

Upon seeing the image of the cartoon character Peppa Pig, the child produces what needs to be understood as a form of mitigated echolalia. According to the child’s mother, the bit on big and small was echoed from the child’s experience at the therapy center, when learning to distinguish sizes and practicing the Mandarin Chinese words for ‘big’ and ‘small’. Note that these two words are integrated into the larger construction ‘X is big, Y is small’, filled with the subject ‘Peppa’, the target image. Hence, the child seems to have produced this mitigated echolalic utterance as a verbal outburst, as a free association that in fact includes the correct name of the image presented.

It should be noted that several echoed enunciations preceding the first question on a stimulus image may not be free associations but instances of anticipatory question-oriented functional echolalia. Halfway through the task, most children seemed to have internalized the pattern and order of questioning. In some cases, such echoed utterances preceding the first question were nevertheless appropriate answers to the test questions. Therefore, many such occurrences might be covert examples of functional question-oriented echolalia. We decided to code such utterances as semi-functional free associations instead, simply because, after examining the recording, we found limited non-verbal indicators (e.g., gaze, body posture, etc.) for us to be absolutely confident of their specific communicative function.

Lastly, two echoed utterances in our data seemed to be used non-functionally upon lengthy discussions with the parents and therapists. These were echoed enunciations that failed to answer the questions posed and neither seemed to manage the conversation nor help the children think. The first example is:

(13) Child 3, Resp. 6: Money bill.
[Responding before question].
Child: 妈妈做饭。
*mama zuo fan.*
Mommy cooks.
Experimenter: 这个可以用来干什么?
*zhe ge ke yi yong lai gan shen me?*
What’s it used for?
Child: 妈妈做饭。
*mama zuo fan.*
Mommy cooks.

In (13), upon seeing the image of the money bill, the child produces a declarative enunciation on his mother cooking that he had previously heard repeatedly, according to his mother. When asked about the function of the money bill, he repeats that echoed utterance again, failing to answer the question. This echoed expression seems semantically irrelevant to the task, as not even the child’s mother could relate the child’s experiences with money with her cooking or the original source of that repetition. Also, the child did not seem to use it to manage the conversation (e.g., express a wish to shift the topic to discuss something related to his mother and/or cooking) or manage his own thoughts (as a means of support towards answering the question). This child also produced this echoed enunciation upon seeing the image of the fruit seller (Resp. 9), for which the mother also saw no connection. One needs money to buy food that one can then cook, so the relation between cooking and the money bill and the fruit seller is closer than if the echoed utterance on cooking had been produced upon seeing the toilet or bed images, for instance. However, the child’s mother was quite certain that this utterance was purely parroted from the child’s earlier verbal experiences. He frequently repeated it in inappropriate contexts in everyday life, making it a candidate for stereotypy.

A similar example by a different child is:

(14) Child 3, Resp. 5: Bed.
[Responding before question].
Child: 妹妹喜欢袋鼠。
*mei mei xi huan dai shu.*
Younger sister loves kangaroos.
Experimenter: 这是什么?
*zhe shi shen me?*
What’s this?
Child: 床上。
*chuang shang.*
On the bed.

Here, the child repeats a line from a cartoon movie, according to his mother. The mother could find no connection between the original utterance and the child’s experience with beds or sleeping. Therefore, this instance was also classified as non-functional echolalia. The child’s later turn shows that he did understand that the target image represented a bed.

There are also ambiguous cases of delayed echolalia in our data. An example is an exact echolalic utterance translated as ‘Smelly. Flushing water, flushing’ (Child 7, Resp. 18), produced before the experimenter’s first question on the toilet, after naming the object twice and providing its function three times. In that context, this could equally be interpreted as topic development (i.e., functional echolalia) or free association (i.e., semi-functional echolalia). Without further evidence, we believe ‘ambiguous’ is the most appropriate designation.

## Quantitative results

Our eight participants with ASD all produced a relatively high proportion of echoed speech. Critically, in our opinion, 120 out of the 196 echolalic instances uttered during the task were unequivocally functional in this study. Most such cases were used to answer the experimenter’s question, while a few occurrences were used to manage the ongoing interaction or the child’s own mental process. We also encountered 58 cases of free associations, constituting semi-functional echolalia, two echoed utterances used for no apparent function, and 14 ambiguous cases.

As for the creativity level of all echoed occurrences irrespective of their functionality, most of them were instances of exact echolalia, only a few instantiating mitigated echolalia. Regarding the span of time between the repetition and the original utterance being echoed, we found a high proportion of delayed echolalia, immediate echolalia making up a very low proportion. As we had expected given the study’s eliciting nature, the highest proportion of these original echoed sources were standard fixed expressions widely recognizable by most or all members of the linguistic community (i.e., socio-communicative formulae and socio-cultural emblems). They were followed by specific prior interactions from the children’s lives only recognized by their close circle, with echoed sources recognizable by a specific group in the community (from cartoon movies, television programs, and storybooks) making the lowest proportion.

In what follows, we present the distribution of: (i) echolalia vis-à-vis referential and descriptive alternatives; (ii) functional, semi-functional, and non-functional echolalia; and (iii) exact vs. mitigated and delayed vs. immediate echolalia, as well as echolalia from different sources.

### The production of echolalia vs. common nouns and statements

In terms of form, the children’s responses were divided into three main categories: echolalia, common nouns, and statements (i.e., creative declarative expressions). The children’s responses to the first question (i.e., ‘What’s/Who’s this?’) were, not surprisingly, mainly either echoed utterances or common nouns, whereas the answers to the second question (i.e., ‘What’s it used for?’ or ‘What does s/he do?’), mainly consisted of either echoed utterances or creative statements. The frequency data of individual participants are shown in Table 4.

**Table 4:** Individuals’ production frequency of echolalia vs. common nouns and statements.

Form-frequency	Common noun	Echolalia	Statement	Other categories	Total
Child 1	69	16	13	21	119
Child 2	46	11	9	3	69
Child 3	28	27	1	9	65
Child 4	43	16	17	3	79
Child 5	29	18	13	5	65
Child 6	43	26	19	6	94
Child 7	68	36	26	6	136
Child 8	49	47	33	2	131
Total	375	197	131	55	758

**Figure 2:**
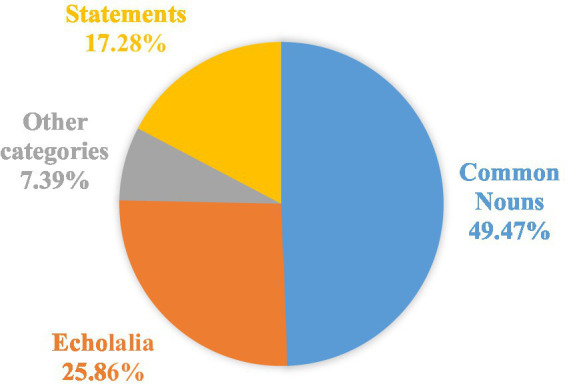
Average distribution of the main formal categories.

The proportions for our eight participants are presented in Figure 2, which encompass all forms and functions (incl. Semi-functional and non-functional echolalia). As Figure 2 presents, participants produced more conventional nouns (almost 50%) than any other categories. However, when compared with the categories of ‘Statements’ and ‘Other’, there was a high proportion of echolalia, the production of verbal formulae being even higher than that of statements (an average of ~26% vs. ~17%). Specifically, echolalia included verbal formulae (e.g., ‘Happy birthday!’) and onomatopoeic occurrences (e.g., ‘beep beep’). Common nouns included concrete common nouns (e.g., ‘teacher’) and family class nouns for politeness (e.g., ‘uncle’, ‘grandmother’, see examples 6 and 9). ‘Statements’ comprise creative non-echoed descriptions of the function or profession depicted therein (e.g., ‘used for singing’ for ‘microphone’, as an answer to the question ‘What is it used for?’).

A few responses could not be categorized as valid –or even interpretable– and were thus excluded from analysis (see Table 5). The first type of such ‘Other’ occurrences was ‘verbal tics’, automatic word strings that were not echoed from a fixed expression or a specific prior enunciation. An example is “*ta hui shuo hua*” (‘She/He can talk’), produced by Child 1 after being asked what a given professional does in as many as 12 of his responses. The child’s mother confirmed that this was not a repetition from the child’s life, and it is not a standard fixed expression in Mandarin Chinese either. Other sub-types in the ‘Other’ category were iconic gestures and creative enactments, namely full one-time theatrical demonstrations produced on the spot, which were thus not echoed. Unrelated or meaningless responses were neither echolalic nor related to the task, and ambiguous cases were those we could not categorize. Lastly, unintelligible responses mostly consisted of mutterings that not even the children’s parents could interpret.

**Table 5:** ‘Other’ category.

Categories	Verbal tics	Iconic gestures	Creative enactments	Unrelated or meaningless	Ambiguous	Un-intelligible	Total
Amount	10	8	1	19	9	9	56
Percentage	1.32%	1.05%	0.13%	2.51%	1.19%	1.19%	**7.39%**

In sum, most occurrences were intelligible and only one evidenced creative enactment, indicating that our participants’ speech was comprehensible but formulaic. It is worth noting that some instances like the ones we categorized as ‘stereotypy’ or ‘ambiguous’ might have been coded as non-functional delayed echolalia by other research protocols without parental or therapist debriefings.

### Functional, semi-functional, and non-functional echolalia

In contrast to the common opinion that echoing may be meaningless and hinder functional language use, our results suggest that echolalia is mostly discernibly functional (~61%). Semi-functional echolalia, which solely involved freely associated verbal formulae and was thus not entirely meaningless or communicatively useless, also made up a relatively high proportion (~30%). Non-functional echolalia constituted by far the smallest proportion of echoed occurrences (~2%). It might be tempting to regard these instances of non-functional echolalia as illustrative of the entire phenomenon, as they stand out from the flow of conversation, but our data suggest the opposite. We must clarify, however, that this is partly a result of the task’s design. Non-functional echolalia may be more frequent in a naturalistic setting when the child is distracted, tired, bored, or distressed. In some cases, it was not possible to determine the specific function of indisputably echoed utterances, even after discussing them at length with the child’s parent (see Table 6).^2^

**Table 6:** Functional, semi-functional, and non-functional echolalia.

Categories	Functional echolalia			Semi-functional echolalia	Non-functional echolalia	Ambiguous	Total
	*Question oriented*	*Conversation oriented*	*Self-oriented*	*Free association*	*Meaningless repetition*		
Amount	92	9	19	58	4	14	196
Percentage	46.94%	4.59%	9.69%	**29.60%**	**2.04%**	**7.14%**	**100%**
	**61.22%**						

Critically, question-oriented functional echolalia encompassed the largest portion, with nearly half of all echolalic utterances (~47%). They were thus mainly used to name the target entity or profession (responses to question one), to describe it (responses to question two), or to expand on a previously introduced topic (developments on prior responses to either question or both). Some echoed occurrences also seemed to function as communicative strategy to manage the conversation (~5%) or as cognitive strategy to help children think (~10%).

As a whole, 8 participants produced 120 echolalic occurrences that are functional, Table 7 shows the frequency of specific functions produced by individual participants. Different from the case for naming, however, participants with a more fluent vocabulary (children 1, 5, 6, 7, and 8) used more echoed verbal formulae as topic development and description.

**Table 7:** The frequency of functional echolalia in individual participants.

Functions-Frequency	Naming	Description	Topic development	Cognitive strategy	Conversation maintenance strategy	Total
Child 1	4	3	7	0	0	14
Child 2	1	2	2	1	0	6
Child 3	6	5	0	0	8	19
Child 4	1	4	0	0	0	5
Child 5	6	5	0	1	0	12
Child 6	0	14	4	2	0	20
Child 7	1	12	3	4	0	20
Child 8	0	7	5	11	1	24
Total	19	52	21	19	9	120

**Figure 3:**
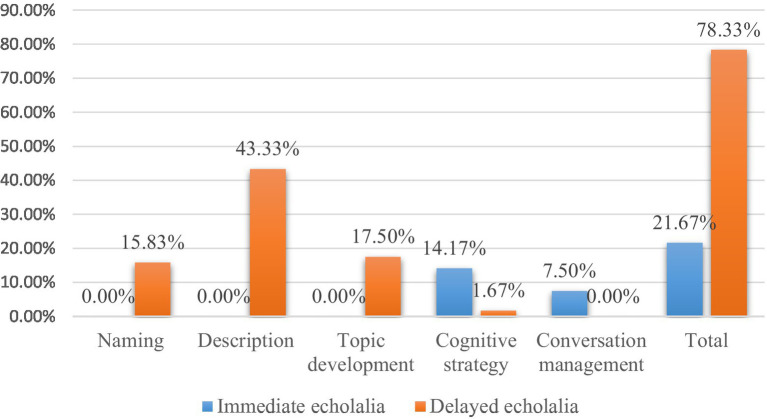
The percentages of total occurrences of echolalia in specific functions.

Figure 3 presents the proportional distribution of echolalia in five specific functions. All question-oriented functions comprised cases of delayed echolalia. Most of these served as descriptions of the professions or the functions of entities. The rest were used to name or expand on an earlier introduced topic. By contrast, all occurrences used as conversational maintenance strategy were of immediate echolalia, while only a small proportion of echolalic utterances were used to hold the floor. Furthermore, all but two occurrences of echolalia used as cognitive strategy consisted of immediate echolalia.

Among these five specific functions, the descriptive function was the most frequently occurring one, making up almost three times the amount of functional echolalia for naming (~43% vs. ~16%, see Figure 3). The second question eliciting more functional echolalia than the first one may indicate that it is harder for children with ASD to produce an entire statement than a common noun. The topic development function was moderately represented, making up a slightly higher proportion than that of naming (~18% vs. ~16%). This is remarkable, relatively small as these proportions and the difference between them are, since ours was not a naturalistic study or one where children were encouraged to talk about their experiences with the referents in the stimuli.

The function of cognitive strategy is the only one that was attained by both immediate and delayed echolalia, even if these were overwhelmingly echoes of the experimenter’s immediately preceding turn. In two cases, these were delayed mitigated repetitions of the task’s previously heard questions before any question on the new image was asked, in which the child integrated the name of the target image (e.g., “*yi sheng zuo shen me yong de?*” ‘What does a doctor do?’, Child 7, Resp. 16; “*hua tong zen me yong de?*” ‘What’s the microphone used for?’, Child 7, Resp. 17). Lastly, the function of communication maintenance strategy constitutes the smallest category, most probably reflecting the nature of the task, with its simple, fixed, and repetitive structure, thereby not inviting conversation shifts.

**Figure 4:**
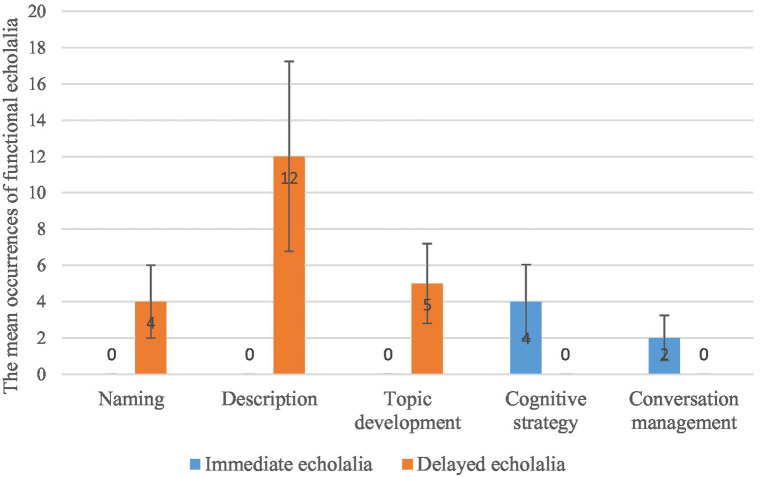
The mean occurrences of echolalia in specific functions.

Additionally, the mean occurrences of echolalia in five specific functions (both immediate and delayed) are presented in Figure 4, in which the error bars display the data’s Standard Errors.

### Time span, creativity level, and sources of echolalia

In this section, we discuss: (i) the time span between echoed occurrences and the original utterances (delayed vs. immediate echolalia); (ii) the level of creativity of echoed instances (exact vs. mitigated echolalia); and (iii) the different sources of echolalia (widely recognizable by the entire linguistic community, by a specific social group, or only by the child’s close circle).

**Figure 5:**
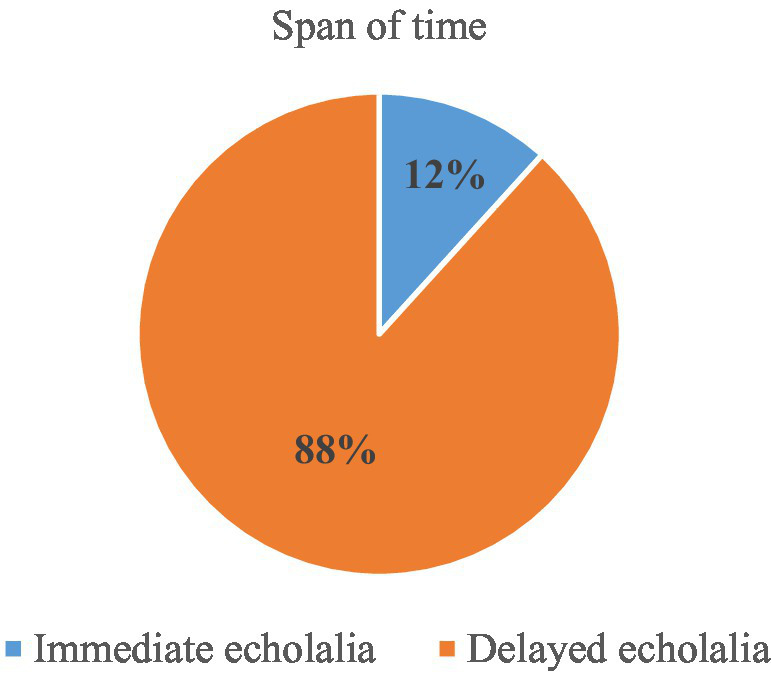
Distribution of immediate vs. delayed echolalia.

The majority of echolalic utterances produced by the children with ASD in this study are delayed ones (see Figure 5). The main reason for this is the use of an elicitation task with only open questions to be answered, which prompted participants to produce new speech grafting onto old speech. Still, the children with ASD in our study uttered a small proportion of immediate echolalia, these mostly being repetitions of the test questions, as exemplified in examples (7) to (10) in the previous section.

**Figure 6:**
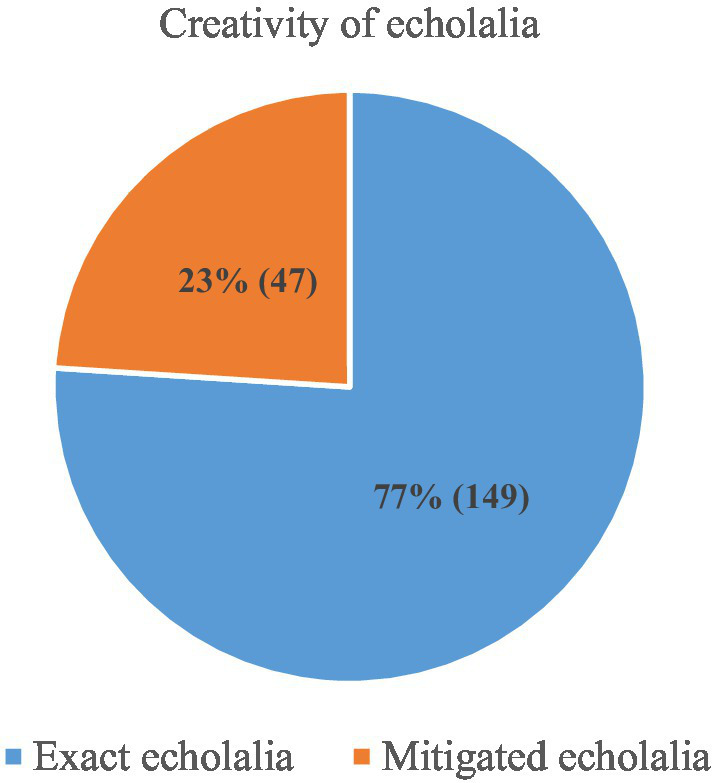
Distribution of exact vs. mitigated echolalia.

Regarding creativity level, we encountered three times more instances of exact echolalia than of mitigated echolalia (see Figure 6). A greater production of exact than mitigated echolalia means that these echoed expressions were entrenched as fixed units of socio-communicative formulae or socio-cultural emblems. Such expressions might be similarly stored as idioms or popular sayings. By contrast, we encountered more mitigated echolalia in specific prior utterances from the child’s own life.

**Figure 7:**
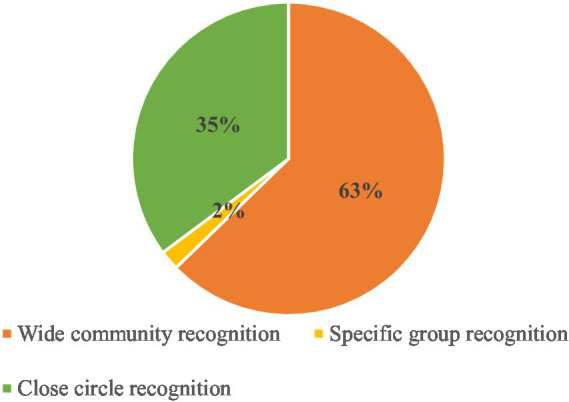
Distribution of the sources of echolalia.

As for the sources of echolalia, most were fixed expressions recognizable by most or all members of the linguistic community (see Figure 7). Specifically, 63% of echolalic occurrences constituted fixed socio-communicative formulae (e.g., “Have some water!” for ‘glass’) or socio-cultural emblems (e.g., “Happy birthday!” for ‘birthday cake’). While our study was designed to elicit easy-to-identify echolalic utterances, 35% of delayed echolalia was specific to a child’s prior verbal experiences. An example is an utterance that translates as ‘her leg was broken, and she stays at the hospital’, produced upon seeing the image of the bed (Child 6, Resp. 14). According to the mother, this was a repetition of her own prior speech, from when a friend of hers broke a leg, and the mother told the child why they were going to visit that friend in the hospital. Lastly, only 2% of echoed occurrences in our data originated in lines from storybooks or cartoon movies (e.g., from ‘Peppa Pig’ and the Ryder character from the ‘PAW Patrol’), recognizable by a specific group in the linguistic community.

These results on the sources of echolalia should be interpreted as partly reflecting our study’s goal of exploring fixed expressions and individual occurrences shared by a large number of speakers in the linguistic community. Thus, the stimuli were all either commonly associated with given standard verbal formulae or with specific prior interactions from popular cartoons. Indeed, in admittedly as small a database, a prior naturalistic study found more examples of echolalic specific prior enunciations than standard socio-communicative or socio-cultural formulae (Pascual et al., 2017).

In these data, functional echolalia made up a relatively high proportion of the overall echolalic occurrences of the entire group, demonstrating that it plays an important role in autism speech. Our participants produced echolalia when failing to retrieve a corresponding noun or generate a creative statement. The cognitive load involved in storing ready-made linguistic units in long-term memory may be much lighter. Hence, functional echolalia may not simply be stereotyped, it may be used as a coping strategy in language pathology.

## Discussion

The elicited echolalic utterances in our dataset show interesting and unexpected forms that had so far not received much attention in the ASD or linguistics literature. An example is echolalia involving multiple viewpoint shifts in one single conversational turn, as in the firefighter example (4), in which the child enacts two fire witnesses and the firefighter himself. Other children also show the ability to shift voices by using verbatim repetitions. Thus, our data show that at least some children with ASD are good at adopting the voice of discourse characters as a means of satisfying an immediate communicative goal, such as to demonstrate the actions of a character or express a character’s response or feeling. The fact that such vocal imitations seem to satisfy local communicative purposes is an important area for future research, as it suggests that viewpoint and viewpoint shifting may not be an all-or-nothing phenomenon. Indeed, some children with ASD selectively imitate and thus take on the viewpoint of characters during familiar question-answer encounters, without necessarily being capable of doing so generally. Our intuition is that Theory of Mind is a complex suite of capacities that include the ability to attribute mental states to others, recognize emotions, and shift perspective and viewpoint (Wellman and Gelman, 1998; Peterson et al., 2005). The imitation of a character’s voice and vocalizations is evidence of viewpoint shifting (which is implied when one takes on a character’s voice), and it appears to be used for local conversational purposes. That said, there remains little doubt that these children still have significant deficits associated with Theory of Mind.

In other cases, the same visual stimulus prompted echoed enunciations ascribed to different conversational participants in the prototypical interaction relative to an elicited semantic frame. For instance, the image of the delivery man led one child to take the voice of the target referent as speaker (e.g., “*ni de bao guo*” ‘[Here are] your parcels’, Child 7, Resp. 5), while another child enacted the customer receiving the package, the target referent being the addressee (e.g., “*xie xie ni!*,” ‘Thank you!’, Child 8, Resp. 5). Our participants thus managed to associate the target stimuli with a whole skeletal semantic frame or script prototypically related to it (e.g., a delivery man). This is striking since children with ASD are well-known for being detailed-oriented and better with grammatical form than with social communication (Tager-Flusberg, 1994, 2001; Naigles, 2017; Naigles and Tek, 2017).

Another interesting phenomenon in our data is the appearance of common nouns embedded in echoed occurrences used for naming. For instance, Child 5 answered the question requesting him to name the towel by using the Mandarin Chinese verb-noun structure “*ca mao jin*” (lit. ‘Wipe towel’, ‘Wipe [my face with] the towel’, Resp. 12), a self-echoed utterance that includes the right name for ‘towel’. According to the child’s mother, every time the child washes his face, she asks him to wipe it with the towel. Thus, the image of the towel prompted this daily washing-up ritual, characterized by an exchange with his mother. Instead of providing the name for ‘towel’ straight away, the child repeats this fixed utterance, which includes the name asked about.

We encountered similar examples in free associations interpretable as answers to anticipated task questions. Two cases include echoing the statements containing the target common noun as the sentence subject, namely “*chang mai ke feng*” (‘Sing [with] the microphone’), produced when presented with the image of the microphone (Child 5, Resp. 17), and “*jing cha you qiang zhua huai ren*” (‘The policeman has guns and catches bad guys’), uttered upon seeing the police officer (Child 8, Resp. 10). Two other instances include echoing the task question with the common noun in the echoed question itself, as in “*yi sheng zuo shen me yong de?*” (‘What does the doctor do?’), related to the image of the doctor (Child 7, Resp. 16), and “*hua tong zen me yong de?*” (‘What’s the microphone used for?’), related to the microphone (Child 7, Resp. 17). This phenomenon has also been identified in spontaneous ASD speech (Dornelas and Pascual, 2016; Pascual et al., 2017). Hence, fixed linguistic units are part of the child’s stored verbal repertoire that may be used as ‘pivot schemas’ for including the common nouns needed to name the concepts with which they are associated, even though nouns are simpler forms. Far from being an impairment, functional echolalia seems to be a template for in-the-moment communicative creativity.

## Conclusion

Most echoed occurrences in our data were functional in this study. All eight participants mastered echolalia for different communicative goals (i.e., naming, description, topic development, conversational maintenance) or thinking aloud (i.e., cognitive strategy). Even when failing to answer the task’s questions in a standard manner, the ultimate meaning and communicative intentions behind their echolalia in that context were clear in most cases in this study. Children with ASD do seem to be aware of social norms and situations; they can associate socio-communicative formulae and socio-cultural emblems within given socio-cultural frames. Echoed verbal formulae, therefore, comprise common effective strategies and are indicators of linguistic and communicative competence in ASD.

These findings are of theoretical and clinical significance, as they indicate the effective use and importance of echolalia in autism speech. While the presence of echolalia may serve a diagnostic function, it is not just pathological but also enabling. Also, functional echolalia, in all forms, not only encompasses immediate echolalia or specific prior interactions from the child’s inner circle, which are the types most studied. Instead, prototypical functional echolalia also includes fixed expressions entrenched in the linguistic community (Laury and Ono, 2020), which should thereby receive more attention in autism research. Given the importance of formulaic linguistic units, manipulated as ‘pre-packaged’ assemblies, in typical language development and ordinary language use (Langacker, 1999; Wray and Perkins, 2000), these should also receive more attention in general and applied linguistic theories.

Moreover, the functional use of echolalic self-talk (e.g., example 10) may pave the way for inner speech. Dialogue is a precursor and product, a mediator and tool of self-system functioning. Dialogue becomes one’s own when appropriated from dialogue with others, their own voices emerging from the voices associated with occupants of social roles. This coincides with what Vygotsky (1962, [1934]) claims is a stage in language development, in which outer dialogue (exchanges with others) precedes internal dialogue (self-talk as cognitive strategy). Also, our evidence of voice shifting in discourse is more consistent with a ‘social–emotional salience approach’ to autism (Weeks and Hobson, 1987; Gaigg, 2012), insofar as the voices taken by the speaker emerge from learning the emotionally salient responses of specific actors in typical situations. Thus, autism does not seem to automatically mean an inability to put oneself in others’ shoes, at least not regarding vocally stereotyped social situations.

Furthermore, since several echolalic utterances were produced before the experimenter’s question, our study participants evidently have forged a strong link between what was represented in the image and their verbal experiences with that referent. This sheds light on how concepts are ‘stored’ in our minds. We not only store the names of concepts (e.g., ‘soccer ball’), our perceptual experience with them (color, shape, size), and simulations of embodied interactions with them (kicking the ball; Barsalou, 1999; Glenberg and Kaschak, 2002; Bergen and Feldman, 2008), but also verbal experiences socio-culturally associated with them (saying or hearing “*Gooal!!*”). Indeed, our data reveal that children with ASD store the target entities together with schematic social frames or scripts prototypically related to them, of which interjected verbal formulae form a salient part. The fact that our participants so often associated the target images with linguistic units commonly related to them in the linguistic community shows that they are good with both association and metonymy. Their anticipating the experimenter’s questions also seems to indicate that autism may not be a disorder of prediction, as has been suggested (Sinha et al., 2014), or not too severely or at all levels.

Clinically, our findings suggest that testing knowledge of and competent use of typical socio-cultural emblems and socio-communicative formulae by individuals with ASD may be important for assessment. These are deeply ingrained in people’s minds and very salient in speech (also by typically developing children, Lieven et al., 1992). This study offers an effective elicitation protocol and visual stimuli which may also be useful for future quantitative studies on partial or verbatim repetitions by children with ASD, as well as for testing individuals with other language pathologies involving echolalia (e.g., aphasia, schizophrenia, semantic dementia). Our protocol and material can be applied to compare a language pathology group with typical groups matched for linguistic competence (e.g., vocabulary, sentence complexity, and mean length of utterance) and/or matched for mental or chronological age, so as to investigate the developmental patterns of echolalia. This study may also inform therapeutic interventions to help children with ASD handle or improve their communicative practices, which should also help caregivers learn how to best interact with them. In sum, the instances of functional echolalia examined here suggest that children with ASD try to communicate and do so in specific circumstances. Echolalia is undoubtedly a symptom of autism, but it may also be the key to effective intervention.

## Data availability statement

The raw data supporting the conclusions of this article will be made available by the authors, without undue reservation.

## Ethics statement

The studies involving human participants were reviewed and approved by Zhejiang University. Written informed consent to participate in this study was provided by the participants' legal guardian/next of kin. Written informed consent was obtained from the individual(s), and minor(s)’ legal guardian/next of kin, for the publication of any potentially identifiable images or data included in this article.

## Author contributions

FX: investigation, study design and interpretation, data collection, analysis, and curation, and writing – original draft, review and editing. EP: investigation, study design, interpretation, data analysis and curation, project administration, and validation, supervision, and writing – review and editing. TO: interpretation, data analysis and writing – review and editing. All authors contributed to the article and approved the submitted version.

## Funding

This research was supported by the National Social Science Foundation of China project “A Contrastive Study of Multimodal Referential Strategies between Children with ASD and their TD Counterparts” (17AYY009) and a ‘Disciplinary and Innovative Project of Zhejiang University’ (106000*194231902), as well as the the ‘Hundred Talents Program for the Humanities’ (411836).

## Conflict of interest

The authors declare that the research was conducted in the absence of any commercial or financial relationships that could be construed as a potential conflict of interest.

## Publisher’s note

All claims expressed in this article are solely those of the authors and do not necessarily represent those of their affiliated organizations, or those of the publisher, the editors and the reviewers. Any product that may be evaluated in this article, or claim that may be made by its manufacturer, is not guaranteed or endorsed by the publisher.

## Footnotes

^1^We follow Pascual et al. (2017) and Dornelas (2018) in including onomatopoeia, as it constitutes a repetition of a previously heard sound. We did not code conventional instances of onomatopoeic words (e.g., for animals), since we are solely interested in echoes. Examples of onomatopoeia coded as echolalic are the imitation of a car’s whistling sound ("*du du ba ba*," Child 2, Resp. 4), a mobile phone’s incoming call ("*ding ding ding*," Child 6, Resp. 22), and the waiting ring ("*du du du*, beep beep beep," Child 2, Resp. 22).

^2^The calculation includes each individual echolalic occurrence rather than only different echoed utterances. For instance, only two echoed utterances were used non-functionally in our data (one on a mother cooking and one on kangaroos), but since the former was uttered twice in example (13) and once related to the fruit seller, we counted four occurrences of non-functional echolalia in total.
